# Cross-Reactivity of Rapid Salmonella Typhi IgM Immunoassay in Dengue Fever Without Co-Existing Infection

**DOI:** 10.7759/cureus.396

**Published:** 2015-12-04

**Authors:** Adnan Bashir Bhatti, Farhan Ali, Siddique Akbar Satti

**Affiliations:** 1 Department of Medicine, Capital Development Authority Hospital, Islamabad, Pakistan

**Keywords:** dengue fever, misdiagnosis, febrile fever, dengue hemorrhagic fever, dengue false positive, dengue cross-reactivity

## Abstract

Introduction: Dengue fever is endemic in developing nations worldwide with as many as 500,000 annual cases of dengue hemorrhagic fever (DHF) and dengue shock syndrome (DSS). A prompt and accurate diagnosis early in the disease course is essential for prompt identification and treatment of severe complications of the dengue virus infection (DVI). We identified cross-reactivity of a rapid IgM test for typhoid fever in patients with febrile illnesses that were determined to be due to dengue virus.

Methods: All patients with documented DVI during a recent epidemic in Pakistan also underwent diagnostic testing for Salmonella enterica serovar Typhi. The diagnosis of DVI was made based on clinical findings and the positive results for dengue non-structural protein 1 antigen (NS1_Ag_) and/or dengue IgM antibody (anti-D IgM) during the acute phase of febrile illness. Patients with positive test results for Salmonella typhi (S. Typhi) IgM also had their blood cultures done.

Results: In the group of 322 patients with clinical and serological evidence of DVI, 107 also tested positive for S. Typhi IgM. Blood cultures were negative for S. Typhi bacteria in all patients. Principal disease features included fever, headache, myalgia, retro-orbital pain, and a rash accompanied by thrombocytopenia and leukopenia. Comparisons of clinical and routine laboratory findings between the S. Typhi-positive and negative groups showed no significant differences. Patients testing positive for both NS1_Ag_ and anti-D IgM were significantly more likely to test positive for S. Typhi* *IgM, even in the absence of typhoid fever. No routine antibiotics were used and all patients survived.

Conclusion: One-third of a large group of patients with primary DVI also demonstrated false positive results for typhoid fever. Cross-reactivity of a rapid immunoassay for typhoid fever has not been previously reported in DVI or any other flavivirus infections. Until these findings can be further evaluated, clinicians should be cautious in interpreting S. Typhi rapid immunoassays and have a high index of suspicion of DVI in dengue fever endemic areas.

## Introduction

The global incidence of dengue virus infections (DVI) has increased dramatically over the past several decades [1-4]. Current worldwide case burden estimate ranges from 20 to 100 million infections annually, including as many as 500,000 cases of dengue hemorrhagic fever (DHF) and dengue shock syndromes (DSS) [4-5]. The annual estimates of DVI-associated mortality exceed 20,000. Other potentially serious infectious organisms that have a similar worldwide endemic distribution include Salmonella typhi (S. Typhi)* *[6]. An accurate diagnosis of DVI is essential in order to identify, as early as possible, those patients at risk for the critical phase of the infection and possible circulatory collapse, shock, and death. It may be difficult to diagnose DVI based only on clinical criteria during the acute phase of febrile Illness. The principal symptoms are non-specific and difficult to distinguish from numerous other febrile illnesses of viral or bacterial origin [7-10]. Thus, patients with febrile illnesses often benefit from specific diagnostic laboratory studies for dengue virus and other candidate infectious organisms endemic to the region [11-12]. In the case of S. Typhi, concurrent dengue fever and typhoid fever are uncommon [11].

During a recent epidemic of dengue fever in Pakistan, febrile patients were tested for DVI by dengue-specific IgM and IgG (anti-D IgM/IgM) immunoassays usually accompanied by dengue non-structural protein 1 antigen (NS1_Ag_) testing. Patients also underwent screening for S. Typhi infection using a rapid immunoassay for S. Typhi-specific IgM/IgG. A number of patients with confirmed DVI were noted also to have positive S. Typhi IgM results despite negative S. Typhi blood cultures. False positive results of rapid immunoassays for antibodies to S. Typhi outer membrane protein in patients with DVI have not previously been reported. Recognizing the risks of an incorrect diagnosis of typhoid fever resulting in delayed treatment of potentially life-threatening complications of DVI, we conducted a more systematic evaluation of the typhoid rapid chromatographic immunoassay in patients with DVI. The Human Ethics Committee of the Capital Development Authority (CDA) Hospital, Islamabad, Pakistan, approved this study.

## Materials and methods

### Patients

Three hundred and twenty-two patients with a diagnosis of primary DVI during a recent epidemic in the Rawalpindi and Islamabad regions in Pakistan between September and December 2012 comprised this study. The medical records of these patients were examined and study data were placed into a master clinical and laboratory database. The clinical data comprised of the findings on the admission history and physical examination, including details of any prior flavivirus infections and signs and symptoms of an acute illness that included high fever and myalgia accompanied by retro-orbital pain, headaches, facial flushing, or a petechial rash. Informed patient consent was obtained at the time of treatment.

### Laboratory studies

All patients had their white blood cell, platelet counts, liver transaminases (AST, ALT), alkaline phosphatase (ALP), and urinalyses test completed. Patients with suspected DVI had serum tested for dengue NS1_Ag_ (RapiGEN Biocredit, Germany) and anti-D IgM/IgM (RapiGEN Biocredit, Germany). Both of these tests are rapid lateral flow chromatographic immunoassays [13-14].

The diagnosis of primary DVI was based on the typical clinical presentation described above and laboratory test results that were positive for NS1_Ag_  and/or anti-D IgM, with negative findings for anti-D IgG. Patients with anti-D IgG on initial screen were excluded from this analysis. Positive anti-D IgM results were confirmed after one week by an ELISA anti-D IgM ELISA kit (Diagnostic Automation/Cortez Diagnostics, Inc., USA). All patients with DVI also were tested for S. Typhi through IgM and IgG chromatographic immunoassay (Typhoid IgG/IgM Rapid Test, Biocan Diagnostics Inc., Canada). A blood culture was done for all patients with positive S. TyphiIgM antibody testing. For the purpose of this study, only patients who were S. TyphiIgM positive and IgG negative were considered in the results. All immunoassays were done according to manufacturer’s instructions with appropriate controls.

### Statistical analysis

Descriptive statistics are expressed as mean (± SD) and comparisons of categorical variables used the Chi-square test or Fisher’s Exact test. Continuous variables in the groups testing positive or negative for S. TyphiIgM were compared using the Student T-test. A p-value ≤0.05 was considered significant.

## Results

A total of 322 patients with DVI comprised this study. The mean age was 37.1 ± 15.6 years (range: 15-70), and there were 126 females (39.1%).

Of the 322 patients, NS1_Ag_ and anti-D IgM/IgG tests were performed on 308 patients and the remaining 15 were only tested for anti-D IgM/IgG antibodies. There were positive results for NS1_Ag _in 255/308 patients (82.7%) and 274/322 (85%) tested positive for anti-D IgM. None of the 322 patients tested for anti-D IgM had anti-D IgG detected. Of all 322 patients with DVI, 107 (33.2%) tested positive for S. TyphiIgM and none of them had positive results for S. Typhi IgG antibodies. Blood cultures were performed in order to check the presence of bacterial infection and all were found to be negative. Clinical characteristics of the S. TyphiIgM-positive and negative groups are compared in Table 1. 

**Table 1:** Principal initial clinical characteristics of 322 patients with primary dengue virus infection (DVI).

	Rapid Typhoid IgM	
	Positive n = 107	Negative n = 215
Gender (m:f)	1.7:1	1.5:1
Age (years)	38 ± 17	36 ± 15
Temperature (F)	102.4 ± 1.6	102.3 ± 1.6
Blood Pressure (mm Hg)		
Systolic (Blood Pressure)	110.1 ± 9.4	110.8 ± 7.1
Diastolic (Blood Pressure)	67.5 ± 9.0	68.3 ± 8.7
Respiratory rate (/min)	13.6 ± 1.4	13.5 ± 1.4
Headache	107 (100%)	215 (100%)
Fever (no. patients %) [mean ± SD]	107 (100%) [102.4 ± 1.63]	215 (100%) [102.4 ± 1.56]
Myalgia	107 (100%)	215 (100%)
Retro-orbital pain	99 (93%)	193 (89%)
Facial flushing	85 (80.2%)	164 (76%)
Petechial rash	39 (37%)	72 (33%)
Conjunctival congestion	23 (22%)	44 (20%)
Nasopharyngeal bleeding	15 (14%)	30 (14%)

Results of laboratory studies according to S. typhi* *groups are summarized in Table 2.

**Table 2:** Principal initial laboratory test results in 322 patients with primary dengue virus infection (DVI). *Study results are mean ± 1SD and (SI mean ± 1SD).

		Rapid Typhoid IgM	
	Normal Values (Range)	Positive n = 107	Negative n = 215
Total WBC count (/μL)* [/10^9^/L]	(4.00-11.0 x 10^3^/μL) [4.00-11.0 x 10^9^/L]	2,700± 880 (2.7 ± 0.88)	2,240 ± 1,120 (2.24 ± 1.12)
Platelet Count (x 10^3/^μL)* [x 10^9^/L]	(150-450 x 10^3^/μL) [150-450 × 10^9^/L]	65.1 ± 46 (65.1 ± 46)	60.4 ± 42 (60.4 ± 42)
Alanine aminotransferase (U/L)* [μkat/L]	(10-40 IU/L) [0.17-0.68 μkat/L]	81.8 ± 22 (1.37 ± 0.37)	81.3 ± 21 (1.36 ± 0.35)
Aspartate aminotransferase (U/L)* [μkat/L]	(10-40 U/L) [0.17-0.68 μkat/L]	89.2 ± 23 (1.49 ± 0.38)	87.6 ± 21 (1.46 ± 0.35)
Alkaline phosphatase (U/L)* [μkat/L]	(36-92 U/L) [0.5-1.5 ​μkat/L]	408 ± 62 (6.81 ± 1.04)	397 ± 68 (6.63 ± 1.14)
Dengue NS1_Ag_ (n)			
Positive result		95 (88.8)	160 (79.6)
Negative result		12 (11.2)	41 (20.4)
Dengue IgM antibody			
Positive result		98 (91.6)	176 (81.9)
Negative result		9 (8.4)	39 (18.1)

Although there were no clinical or routine laboratory findings that distinguished the groups, cross-reactivity of the S. typhiIgM test was significantly associated with the presence of anti-D IgM antibodies. Those patients with positive results for both NS1_Ag _and anti-D IgM were significantly more likely to have a false positive test for S. typhi IgM compared to patients testing negative for both (p<0.001) (Table 3).

**Table 3:** Probabilities of false positive S. typhi IgM in dengue virus infection (DVI) by rapid immunoassay.

	Typhoid IgM POS GROUP n (%)	Typhoid IgM NEG GROUP n (%)	Fisher Exact P value
NS1_Ag_ Positive	95 (88.8)	160 (79.6)	0.0563
NS1_Ag_ Negative	12 (11.2)	41 (20.4)	
Dengue IgM Positive	98 (91.6)	176 (81.9)	0.0206
Dengue IgM Negative	9 (8.4)	39 (18.1)	
Both NS1_Ag_ and Dengue IgM Positive	100 (93.5)	155 (77.1)	0.0002
Both NS1_Ag_ and Dengue IgM Negative	7 (6.5)	46 (22.9)	

All patients were treated with paracetamol and parenteral fluids. Forty-nine patients required platelet transfusions for profound thrombocytopenia and/or bleeding. No antibiotics were administered for the false positive culture negative S. typhi tested patients.

## Discussion

The diagnosis of the acute phase of DVI during the febrile phase was based on clinical presentation with confirmatory testing with dengue NS1_Ag_ and dengue-specific IgM antibodies in most patients. During the acute phase, the combined uses of these tests provide reliable results for all dengue virus serotypes [9-15] with reported sensitivities and specificities of 89% and above [13, 16-17].

Likewise, contemporary rapid immunoassays for typhoid fever combined with blood cultures for S. Typhi have enabled the timely diagnosis of typhoid fever, particularly in developing nations [18]. To the best of our knowledge, the cross-reactivity in typhoid negative dengue fever has not been described to date.

Early in the course, symptoms of DVI are generally nonspecific, usually characterized by several days to one week of high fever and often accompanied by a headache, retro-orbital pain, malaise, myalgia, and arthralgia. At this phase of illness, it is clinically difficult to distinguish DVI with confidence from numerous other infectious illnesses, including S. Typhi. However, making an accurate diagnosis during the acute phase of the febrile illness is essential for prompt intervention since it is difficult to predict whether the patient will progress to the critical phase characterized by a rapid onset of a severe capillary leak, circulatory collapse, and hemorrhage with thrombocytopenia. These patients require hospitalization and support with meticulous fluid and, when necessary, platelet infusions.

Based on the findings in this study, patients in the febrile phase of DVI may be at risk for misdiagnosis of typhoid fever based on the cross-reactivity of a rapid immunoassay for anti-S. TyphiIgM antibodies. Delays in rapid diagnosis of DVI can be life-threatening. The potential impact of inaccurate diagnostic testing using relatively low cost, rapid assays is considerable since the incidence of DVI worldwide is rapidly increasing [19], particularly in developing nations, including Pakistan, where seasonal dengue epidemics are common. In many of these same regions, typhoid fever is endemic. This greatly increases the risk of misdiagnosis of DVI as typhoid.

It is highly likely that our patient sample had primary DVI and not a secondary infection since circulating dengue NS1_Ag_ was identified in almost all cases accompanied by anti-D IgM but no IgG antibodies. In a group of patients with DVI, sensitivity and a positive predictive value of 97.3% and 100%, respectively, was observed for NS1Ag when NS1 antigen-capture ELISA was used [13].. In other studies, the combination of NS1_Ag_ and anti-D IgM immunoassay testing have similar performance when combined [17]. Although IgM also may appear early in secondary infections, dengue-specific IgG usually appears early as well, as an amnestic response regardless of the infecting dengue serotype [12]. It is highly unlikely that the patients testing positive with the IgM rapid test for typhoid fever represented a group with dengue virus and S. Typhi co-infection. Although such patients have previously been reported, the patients in our study had negative blood cultures and recovered without the routine use of antibiotics.

There are several types of tests for DVI that required different levels of clinical laboratory expertise and resources. The diagnosis of DVI can be done by viral culture, molecular techniques, or immunospecific testing for the presence of the dengue antigen or anti-dengue IgM and IgG antibodies in the blood. Viral culture provides confirmatory evidence of infection but is not useful for early diagnosis. Reverse transcription polymerase chain reaction (PCR) for dengue has a high sensitivity and specificity, but such testing requires laboratory expertise and resources not available in many of the endemic areas. Test kits for DVI that do not require high levels of technical resources include NS1_Ag _and anti-D IgM/IgG antibodies [20-21]. There are no reports of cross-reactivity of these immunoassay kits with S. Typhi. However, the common use of NS1Ag, anti-D IgM/IgG, and anti-S. Typhi IgG/IgM diagnostic kits in developing nations where typhoid fever is also common greatly increases the risk of false positive testing for typhoid fever in patients with DVI. Our findings should be cautiously taken into account until further studies confirm this observation in other dengue virus outbreaks. However, clinicians and clinical laboratory personnel must be aware that a positive anti-S. Typhi IgM does not exclude the possibility of a DVI, nor is it sufficient to conclude that the illness is due to a co-infection.

## Conclusions

We report false positive (cross-reactivity) results for S. Typhi* *serum IgM by rapid chromatographic immunoassay in one-third of patients during the febrile phase of DVI. Physicians should be aware of this cross-reactivity in endemic or epidemic areas of dengue virus to prevent life-threatening complications of DVI. There should be a high index of suspicion of DVI in patients who present with positive S. Typhi IgM by rapid immunoassay. Treatment focused on typhoid fever due to cross-reactivity of tests may preclude the timely diagnosis and intervention of life-threatening complications of DVI.
